# Giant Bullous Emphysema Mimicking Spontaneous Pneumothorax

**DOI:** 10.7759/cureus.31182

**Published:** 2022-11-07

**Authors:** Rudra P Samanta, Srikant Agarwal, Susmita Sengupta

**Affiliations:** 1 Department of Pulmonology, Tata Main Hospital, Jamshedpur, IND

**Keywords:** emphysema, tension bulla, pneumothorax, dyspnea, giant bullae

## Abstract

Emphysema is a progressive and degenerative lung disease that most commonly occurs due to many years of smoking or exposure to smoke and irritants. It is also seen in the congenital absence of the alpha-1-antitrypsin enzyme. Bullous emphysema is an advanced stage of the disease where strictures of the bronchi permit the inspired air to enter the bronchi but close on expiration, causing air retention and alveolar dilation, destruction, and atrophy. Multiple small bullae coalesce to form a giant bulla (defined as occupying more than one-third of the hemithorax), which causes respiratory symptoms and mediastinal shifting and leads to a poor general condition of the patient. Here, we present the cases of two patients diagnosed with bullous emphysema who presented within three months of each other. This article details the similarities and differences in the approach to both cases and the learning experience from these presentations, especially in acute symptomatology. Bullous emphysema is usually confused with a pneumothorax on a simple chest X-ray; hence, it is imperative to look for the lung margins and confirm the diagnosis using computed tomography of the thorax.

## Introduction

Emphysema is a condition when there is an abnormal, permanent enlargement of air spaces distal to the terminal bronchioles of the lungs accompanied by the destruction of the alveolar walls and presenting without obvious fibrosis. The pattern of airspace enlargement is not uniform; hence, the orderly appearance of the acinus and its components is disturbed and usually lost. A bulla is defined as an airspace greater than 1 cm in diameter surrounded by normal parenchyma. A giant bulla is defined as when the bullae occupy more than one-third of a hemithorax [1,2]. A tension bulla is a giant bulla whose size increases due to a check valve mechanism wherein air enters inside the alveoli but cannot exit [3]. Normally, bullae are associated with emphysema but may also be seen in conditions such as asthma and bronchiectasis [3]. Here, we present two cases where giant bullae were misdiagnosed as a case of pneumothorax, and inadvertently an intercostal chest tube (ICT) was placed in one of the cases, while in the second case computed tomography (CT) of the thorax was able to differentiate between the two entities before an ICT could be placed. CT thorax is essential in differentiating between giant bullae and pneumothorax and should be done before specific treatment protocols are initiated.

## Case presentation

Case one

**Figure 1:**
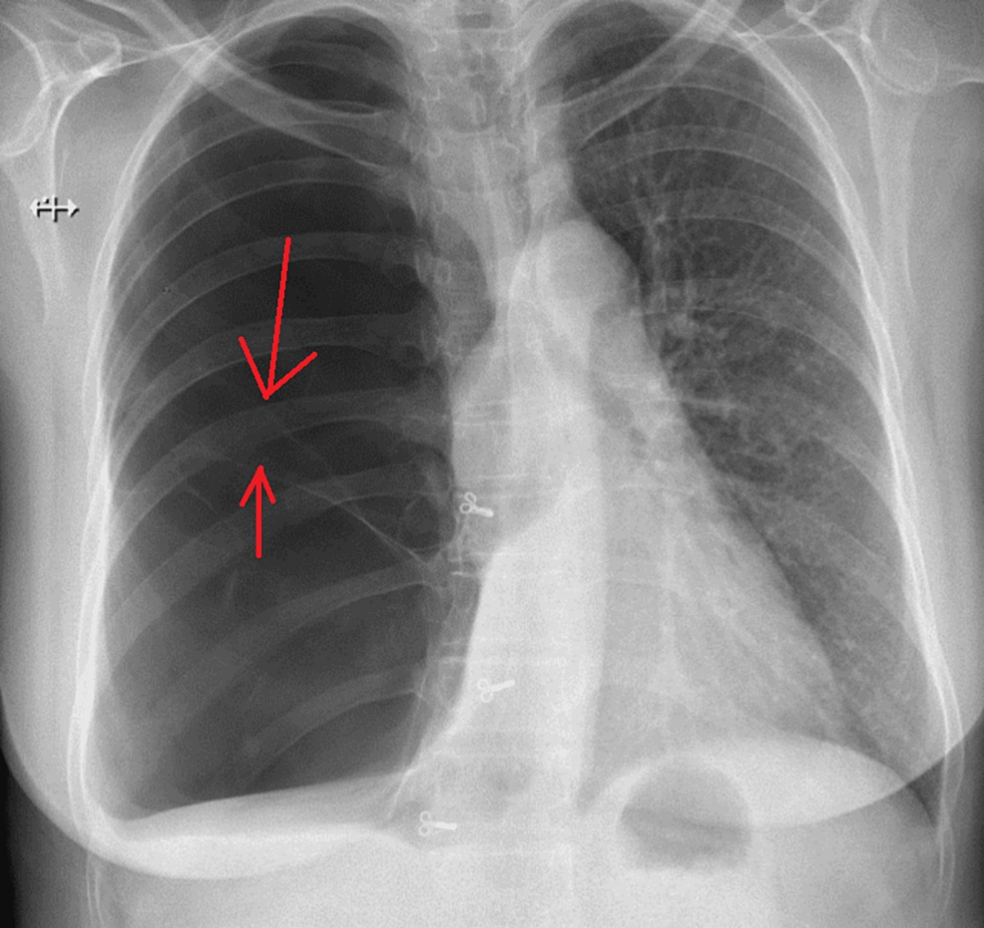
Chest X-ray posteroanterior view on admission. Image showing hyperlucent right lung with no pleural line, mediastinal shift to left, and the presence of irregular lines which indicate walls of the bullae (thin red arrows).

A 48-year-old female presented with increased dyspnea (modified Medical Research Council (mMRC) grade II), right-sided chest pain, and generalized weakness for eight to nine days, without any other associated symptoms. There was no history of medical illnesses such as bronchial asthma, chronic obstructive pulmonary disease (COPD), or bronchiectasis. The patient was a non-smoker. On admission, physical examination was normal, and her vitals were within normal limits with an oxygen saturation of 96% on room air. An examination of the respiratory system showed the trachea deviated to the left, the use of accessory muscles of respiration, decreased vocal fremitus, and hyper-resonance on the right side. On auscultation of the chest, vesicular breath sounds were heard bilaterally with decreased intensity on the right side. A cardiac examination revealed normal heart sounds. Chest X-ray showed a right-sided hyperlucent lung, with a pleural line not evident, and mediastinal shifting toward the left (Figure 1).

**Figure 2:**
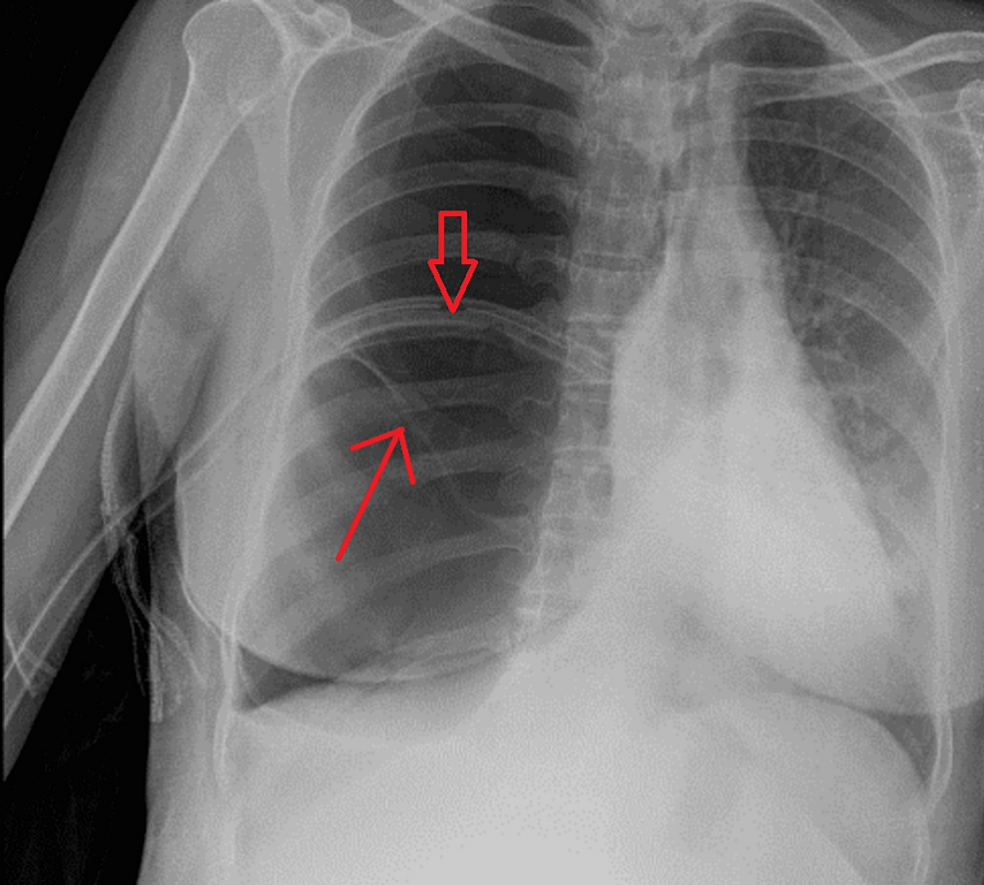
Chest X-ray after ICT insertion. Image showing persistent hyperlucency with no change compared to the previous X-ray; the wall of bullae seen (thin red arrow), and in-situ ICT seen (wide red arrow). ICT: intercostal chest tube

In view of the radiological suspicion of pneumothorax by the emergency physician, an intercostal chest tube (ICT) was inserted in the right pleural space. Post-ICT chest X-ray showed persistent right-sided hyperlucent lung, similar to the pre-ICT chest X-ray (Figure 2).

**Figure 3:**
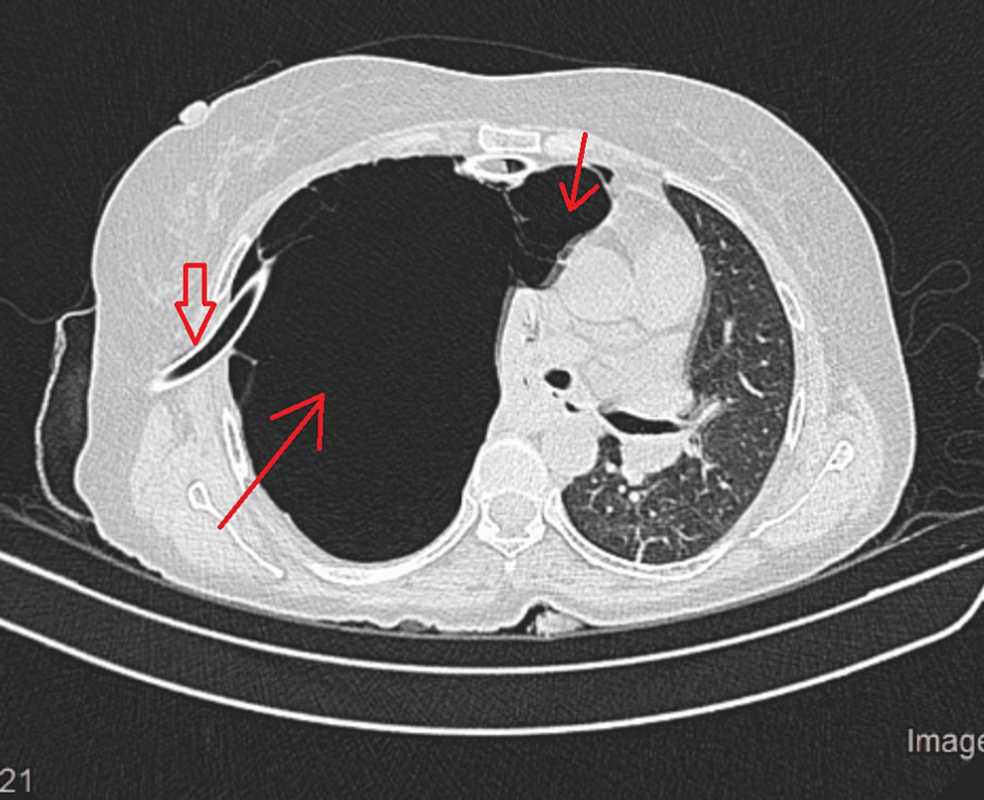
CT of the thorax after ICT insertion. Image showing multiple large giant bullae (thin red arrows) completely occupying the right hemithorax with mass effect in the form of a mediastinal shift toward the left side, with anterior, trans-mediastinal herniation. The residual pulmonary parenchyma is collapsed, with the ICT seen in situ (wide red arrow). CT: computed tomography; ICT: intercostal chest tube

A contrast-enhanced computed tomography (CECT) of the thorax was done to rule out other differentials and check for non-resolution of pneumothorax which showed large giant bullae completely occupying the right hemithorax with mass effect in the form of a mediastinal shift toward the left side, with anterior, trans-mediastinal herniation, and there was no evidence of any focal mass lesion. The residual pulmonary parenchyma appeared collapsed with the ICT tip lying anteriorly in the pleural space (Figure 3).

The ICT was clamped for two days continuously, and during that period, there was no change in the dyspnea status nor the chest pain; hence, the ICT was subsequently removed. In the six-minute walk test, 307 m were covered in six minutes, and her baseline oxygen saturation was 96%, with the lowest recorded saturation being 90%. Spirometry showed the ratio of forced expiratory volume in the first second (FEV1) to forced vital capacity (FVC) to be 0.54, FEV1 was 3.02 L (70% of predicted), and her FVC was 5.6L (78% of predicted). Spirometry showed an obstructive ventilatory defect with no significant reversibility after the administration of bronchodilators. The patient was managed conservatively with bronchodilators (inhaled tiotropium) and discharged.

Case two

**Figure 4:**
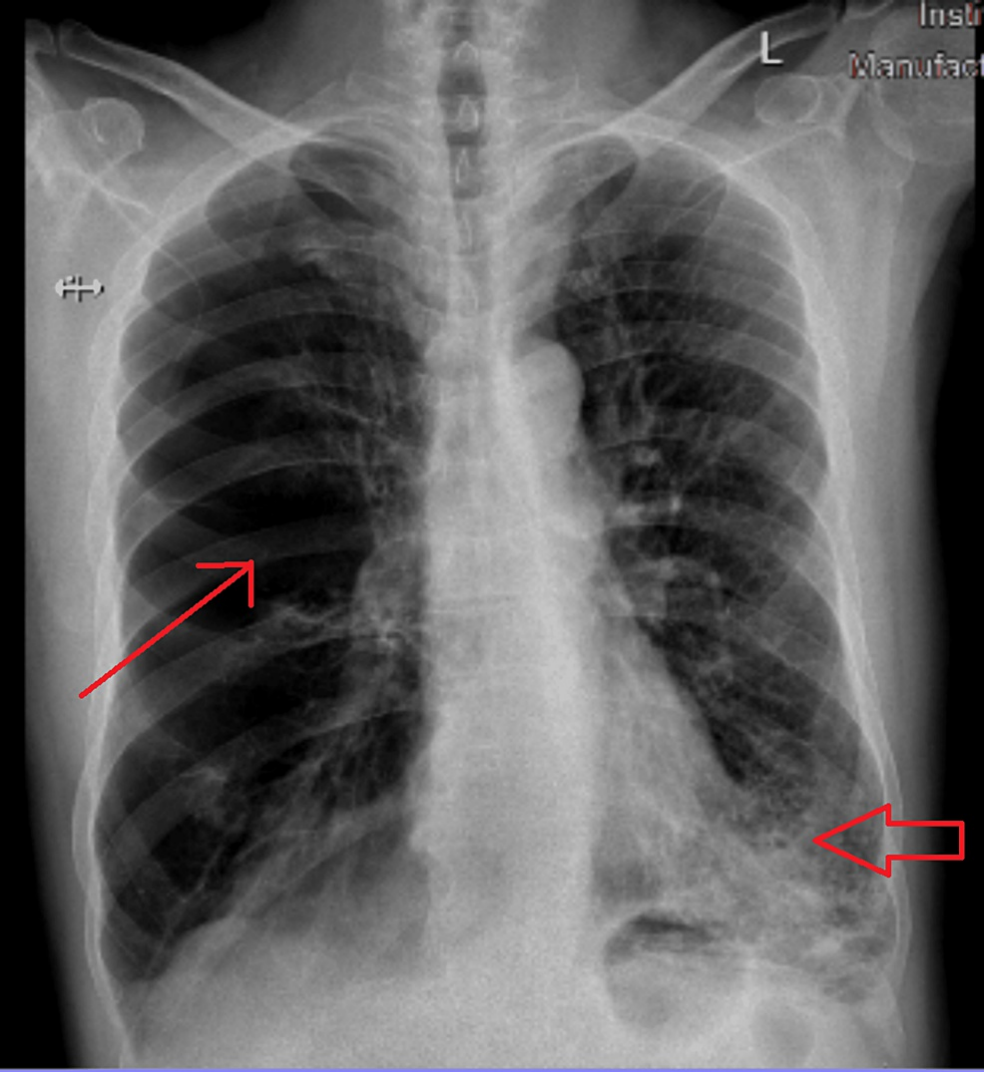
Chest X-ray on admission. Image showing right hyperlucent lung (thin red arrow) with no pleural line and left lower zone fibrotic opacities (wide red arrow).

A 61-year-old male presented to the emergency department, approximately three months after the previous patient, with productive cough and breathlessness (mMRC Grade III). He was a known case of COPD on long-term medication. The patient was a chronic smoker with a smoking history of 30 pack-years but had stopped smoking for the last 10 years. His general examination was normal. An examination of the respiratory system showed increased use of accessory muscles of respiration, decreased vocal fremitus, and hyper-resonance on the right side. On auscultation of the chest, the right side had reduced air entry, with vesicular breath sounds bilaterally. A cardiac examination revealed normal heart sounds. His arterial blood gas report was normal on ambient air. His chest X-ray showed bilateral lower lobe fibrosis and hyperlucency of the right lung, with mild mediastinal shifting to the left (Figure 4).

In the six-minute walk test, 255 m were covered over six minutes. Baseline oxygen saturation was 96%, with the lowest recorded saturation being 85%. Spirometry revealed a very severely obstructive ventilatory defect with no significant reversibility after the administration of bronchodilators. Spirometry was done which showed the ratio of FEV1 to FVC to be 0.45, FEV1 was 1.9 L (20% of predicted), and FVC was 4.24 L (45% of predicted).

**Figure 5:**
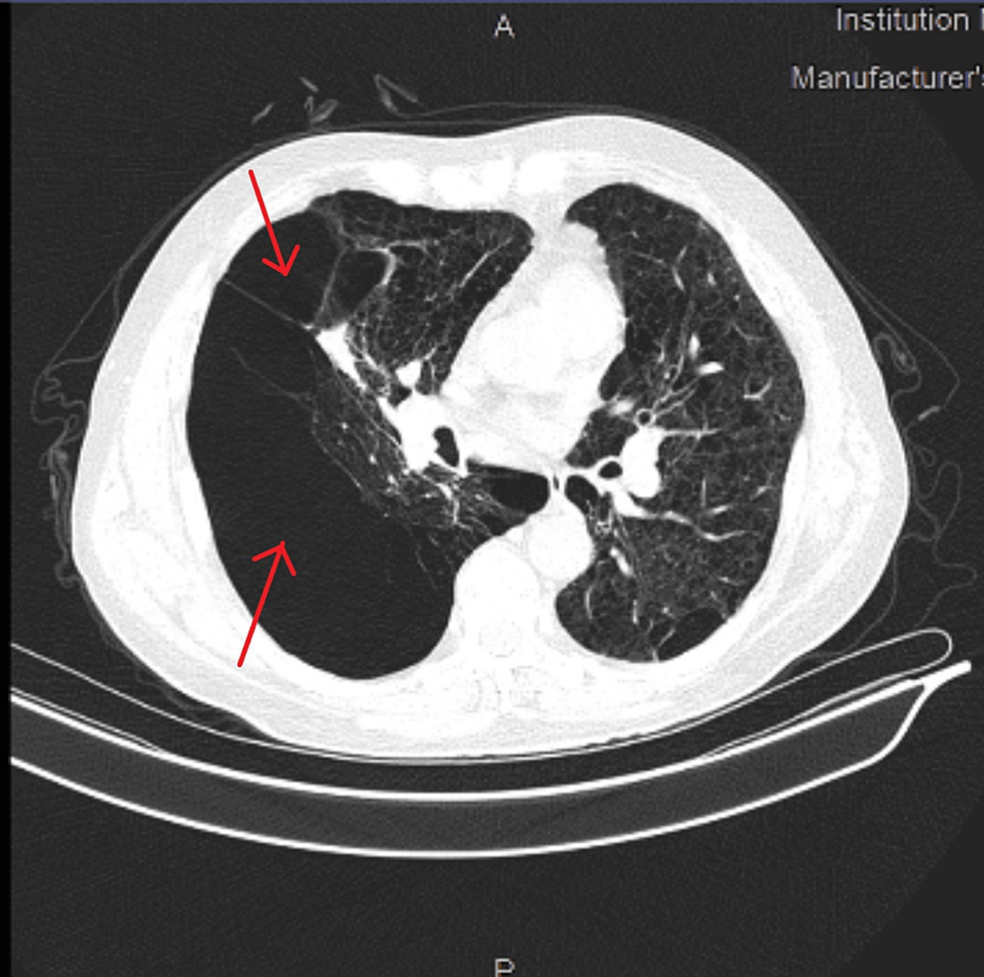
HRCT of the thorax. Image showing diffuse panlobular emphysematous changes along with multiple large bullae involving the right lung (thin red arrows) and a mediastinal shift toward the left side, as well as diffuse paraseptal and confluent centrilobular emphysematous changes along with bullae in the left lung.

A high-resolution CT of the thorax was performed which showed diffuse panlobular emphysematous changes involving the right lung field with associated hyperinflation and mild mediastinal shift toward the left side, as well as diffuse paraseptal and confluent centrilobular emphysematous changes involving the left lung (Figure 5).

The patient was managed as a case of COPD with acute exacerbation symptomatically with nebulized bronchodilators and steroids, oxygen inhalation, antibiotics, and antihypertensive medications.

Both patients did not opt for surgical intervention due to their poor financial condition.

## Discussion

Giant bullous emphysema, as described by Burke in 1937 [4], is a distinct clinical syndrome without any known cause characterized by progressive dyspnea and extensive, asymmetric predominantly upper lobe bullous emphysema which ultimately progresses to respiratory failure. Radiologically, according to Sharma et al. [5], giant bullous emphysema is defined as the presence of giant bullae in one or both upper lobes, occupying at least one-third of the hemithorax and compressing the adjacent normal lung.

Radiologically, bullae appear as radiolucent avascular areas with thin curvilinear walls which are usually less than 1 mm in thickness and thus may not be visible sometimes on a chest X-ray which makes it difficult to detect and leads to misdiagnosis as a pneumothorax. The wall of the bullae when visible may sometimes be misinterpreted as a pleural line and can be confused with pneumothorax on a chest X-ray. To differentiate between a pneumothorax and a giant bulla, the double wall sign is used, which is seen on a CT of the thorax when there is air on both sides of the bulla wall parallel to the chest wall. The absence of this sign rules out pneumothorax; hence, careful viewing can prevent further mishaps and iatrogenic injuries due to inadvertent ICT insertion in a patient with bullous disease. The size, distribution, and locations of bullae on high-resolution CT are predominantly subpleural or intraparenchymal [6]. Moreover, it is important to note that two adjacent bullae in a CT can produce what is known as a false or apparent double wall sign, mimicking a pneumothorax. It can be avoided by viewing closely multiple images that show the absence of air in the pleural space and that the bulla wall is not parallel to the chest wall or parietal pleura [7,8]. Pneumothorax is a serious complication in patients with an already compromised lung function and in those with a prior respiratory disease history which requires immediate placement of an ICT. Therefore, distinguishing carefully between bullae and pneumothorax is important to avoid iatrogenic pneumothorax or bronchopleural fistulae in patients with bullous disease.

Large bullae presenting without symptoms are usually treated conservatively or managed with bronchoscopy with the placement of bronchial valves or lung volume reduction coils. The indications of bullectomy are scarce. Bullae usually progress gradually, but there are some cases that report the spontaneous resolution of giant bullae [9]. Bullectomy, however, is the mainstay of treatment for tension bullae. Patients with non-functioning bullae which compress normal tissue and occupy space in the chest cavity benefit the most from surgical procedures. Giant bullae can be removed surgically in case of an increasing size of bullae, pneumothorax, respiratory distress, hemoptysis, and infection in bullae. A better post-surgical result and prognosis are obtained in patients without underlying lung disease.

## Conclusions

Both cases presented here spell out the importance of distinguishing between giant bullae, tension bulla, and pneumothorax, especially before placing an ICT. Chest X-rays sometimes can be difficult in differentiating these entities. A CT of the thorax is more helpful, especially if there is the presence of a double wall sign. Close viewing of CT images can be helpful in differentiating the double wall sign from the apparent double wall sign. The correct diagnosis can prevent iatrogenic mishaps and determine the care plan and management accordingly.
